# InDel Marker Based Estimation of Multi-Gene Allele Contribution and Genetic Variations for Grain Size and Weight in Rice (*Oryza sativa* L.)

**DOI:** 10.3390/ijms20194824

**Published:** 2019-09-28

**Authors:** Sadia Gull, Zulqarnain Haider, Houwen Gu, Rana Ahsan Raza Khan, Jun Miao, Tan Wenchen, Saleem Uddin, Irshad Ahmad, Guohua Liang

**Affiliations:** 1 Jiangsu Key Laboratory of Crop Genetics and Physiology/Co-Innovation Center for Modern Production Technology of Grain Crops, Key Laboratory of Plant Functional Genomics of the Ministry of Education, Yangzhou University, Yangzhou 225009, China; 2 Rice Breeding and Genetics Section, Rice Research Institute, Kala Shah Kaku, Lahore, Pakistan; 3 Key Laboratory of Genetics and Breeding in Forest Trees and Ornamental Plants, College of Biological Sciences and Technology, Beijing Advanced Innovation Center for Tree Breeding by Molecular Design, National Engineering Laboratory for Tree Breeding , Beijing Forestry University, Beijing 100083, China; 4 Joint International Laboratory of Agriculture and Agri-Product Safety, Yangzhou University, Yangzhou 225009, China

**Keywords:** rice (*Oryza sativa* L.), grain size and weight, Insertion/Deletion (InDel) markers, multi-gene allele contributions, genetic variation, rice germplasm

## Abstract

The market success of any rice cultivar is exceedingly dependent on its grain appearance, as well as its grain yield, which define its demand by consumers as well as growers. The present study was undertaken to explore the contribution of nine major genes, *qPE9~1*, *GW2*, *SLG7*, *GW5*, *GS3*, *GS7*, *GW8*, *GS5*, and *GS2*, in regulating four size and weight related traits, i.e., grain length (GL), grain width (GW), grain thickness (GT), and thousand grain weight (TGW) in 204 diverse rice germplasms using Insertion/Deletion (InDel) markers. The studied germplasm displayed wide-ranging variability in the four studied traits. Except for three genes, all six genes showed considerable association with these traits with varying strengths. Whole germplasm of 204 genotypes could be categorized into three major clusters with different grain sizes and weights that could be utilized in rice breeding programs where grain appearance and weight are under consideration. The study revealed that TGW was 24.9% influenced by GL, 37.4% influenced by GW, and 49.1% influenced by GT. Hence, assuming the trend of trait selection, i.e., GT > GW > GL, for improving TGW in the rice yield enhancement programs. The InDel markers successfully identified a total of 38 alleles, out of which 27 alleles were major and were found in more than 20 genotypes. GL was associated with four genes (*GS3*, *GS7*, *GW8*, and *GS2*). GT was also found to be regulated by four different genes (*GS3*, *GS7*, *GW8*, and *GS2*) out of the nine studied genes. GW was found to be under the control of three studied genes (*GW5*, *GW8*, and *GS2*), whereas TGW was found to be under the influence of four genes (*SLG7*, *GW5*, *GW8*, and *GS5*) in the germplasm under study. The Unweighted Pair Group Method with Arithmetic means (UPGMA) tree based on the studied InDel marker loci segregated the whole germplasm into three distinct clusters with dissimilar grain sizes and weights. A two-dimensional scatter plot constructed using Principal Coordinate Analysis (PCoA) based on InDel markers further separated the 204 rice germplasms into four sub-populations with prominent demarcations of extra-long, long, medium, and short grain type germplasms that can be utilized in breeding programs accordingly. The present study could help rice breeders to select a suitable InDel marker and in formulation of breeding strategies for improving grain appearance, as well as weight, to develop rice varieties to compete international market demands with higher yield returns. This study also confirms the efficient application of InDel markers in studying diverse types of rice germplasm, allelic frequencies, multiple-gene allele contributions, marker-trait associations, and genetic variations that can be explored further.

## 1. Introduction

Rice is the most consumable food commodity in the world and is used as a staple by more than 50% of the world’s population [1]. Rice is also the third-highest produced agricultural commodity after sugarcane and maize [2]. It is a highly valuable grain crop with regard to human nutrition, as well as caloric intake, which provides more than one-fifth of the calories being consumed by humans worldwide [3]. The rapid increase in the human population is further boosting its demand. In some countries, rice is the only staple food, whereas in some other countries, rice is consumed as a traditional dish, as well as an important ingredient in different dishes. In the international market, quality of rice grain is highly indicative of its price, which also reflects its sale-ability. Therefore, both yield and grain quality are equally important parameters for varietal improvement in rice breeding programs. 

**Figure 1:**
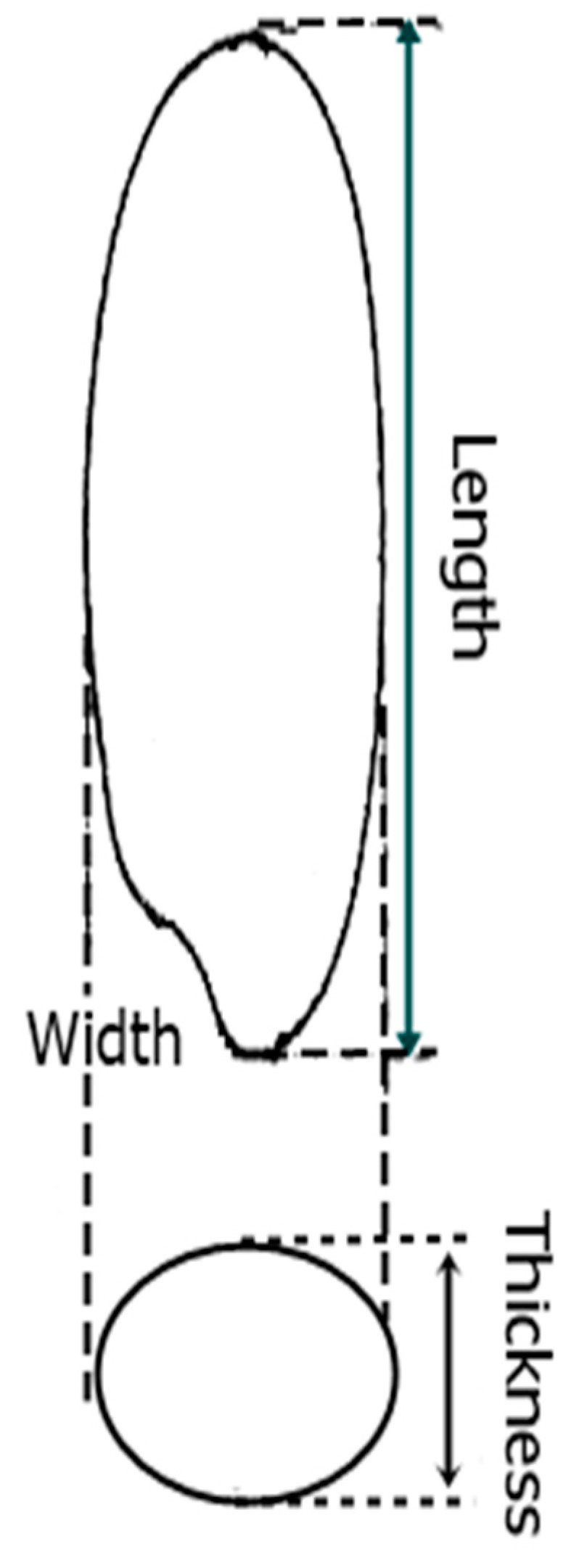
Longitudinal and radial-pattern cross section diagrams of rice grain showing the grain length (GL), grain width (GW), and grain thickness (GT).

Commercial success of a modern rice cultivar is highly dependent on grain size related traits (e.g., grain length, grain width, and thickness) for quality and grain weight associated traits (most importantly thousand grain weight) for grain yield [4]. Grain size related traits determine the rice’s final market value as defined by consumer preferences, which are a combination of grain size, length, and thickness (Figure 1). Some consumers prefer long grains, while some prefer short and bold grains. Likewise, grain yield is determined by three major components, including grain weight, panicles per plant, and grains per panicle. Among these, the most associated trait is grain weight which is determined as the 1000 grain weight. Therefore, grain length, width, and thickness along with the 1000 grain weight, are central benchmarks for breeding grain appearance, as well as yield improvement, in rice. However, due to the quantitatively inherited nature of these traits, breeders hardly rely on phenotypes for their improvement [5]. Therefore, the use of genetic markers is considered superior to phenotyping [6], because such markers are not affected by the environment and are more efficient and reliable compared to phenotypic data. 

A number of Quantitative Trait Loci (QTLs) for grain appearance and weight have already been investigated and reported by different scientists [7,8,9,10,11,12,13,14,15,16,17,18,19,20]. More often, grain length, thickness, and width are regarded as determinants of grain appearance whereas the 1000 grain weight determines grain weight and eventually grain yield. As reported, these traits are under the control of several or many genes and are highly influenced by environmental factors. So far, several major QTLs influencing grain appearance and grain weight have already been characterized and investigated by many researchers. These major genes/QTLs include *qPE9~1* [17], *GW2* [12], *SLG7* [21,22], *GW5* [23,24,25,26], *GS3* [27,28,29], *GS7* [30], *GW8* [31], *GS5* [32], and *GS2* [33,34,35,36]. 

*GS2* (*GRAIN SIZE 2*), also reported as *GL2* (*GRAIN LENGTH 2*) or *PT2* (*PANICLE TRAIT 2*), is a rare allele directly controlling two important grain size related traits, including grain width and grain length in rice. *GS2* is found to encode a transcriptional regulator protein named Growth-Regulating Factor 4 (OsGRF4), which is then targeted by OsmiR396, which is a microRNA causing termination of the OsGRF4 function. Several studies have shown that a 2 bp substitution mutation in *GS2* disturbs the binding of OsmiR396 on OsGRF4, resulting in its overexpression, which in turns increases cell enlargement and enhances cell division in grains, causing longer and wider rice grains [33,34,35,36]. *GS3* (GRAIN SIZE 3) was among the first reported genes to have minor effects on grain thickness and width. In published studies on *GS3*, the *GS3* was demonstrated to be a negative regulator of grain size, and its encoded putative transmembrane protein contains a plant-specific organ size regulation (OSR) domain as a negative regulatory motif, whose function is inhibited by its tumor necrosis factor receptor/nerve growth factor receptor (TNFR/NGFR) family, cysteine-rich domain and von Willebrand factor type C (VWFC) domain. All four domains have been reported to regulate cell divisions in the upper epidermis of the glume inside the rice seed, causing minor effects on the cell size [29]. 

*GS5* (*GRAIN SIZE 5*) has been reported by many researchers who described this gene as a regulator of grain filling and weight. *GS5* promotes cell division in rice seed and, to some extent, elongation of the cells located in the lemma and palea [32]. The encoded protein of *GS5* (i.e., putative serine carboxypeptidase) executes its function as a positive regulator of a subset of the transition genes (G1-to-S) of cell cycle, causing increased cell divisions and resulting in enhanced grain filling and grain weight. Likewise, the *GW2* (*GRAIN WIDTH 2*) gene encodes a protein (RING-type) that has E3 ubiquitin ligase activity, which degrades the ubiquitin–proteasome pathway. *GW2* negatively regulates cell division by suppressing its substrate(s) to proteasomes for regulated proteolysis. The absence or loss of the *GW2* function via its mutation causes enhanced milk filling in grains and enlarged endosperm cells, resulting in a wider spikelet hull [12].

*GW5* (*GRAIN WIDTH 5*), also reported as *SW5* (*SEED WIDTH 5*) and *GSE5*, was investigated by many researchers [23,24,25,26], who discovered that *GW5* is negatively associated with rice grain width and weight. Later, it was revealed that *GW5* actually encodes a calmodulin-binding protein, and *GW5* physically interacts with calmodulin AsCaM1-1, which is responsible for grain width in rice. The deletion of *GW5* or its mutations result in wider grains, indicating its negative effects on grain width. Likewise, *SLG7* (*GRAIN LENGTH 7*), also known as *GW7* (*GRAIN WIDTH 7*), has been identified to encode a TONNEAU1-recruiting motif protein responsible for increased cellular division in the longitudinal direction and reduced cell division in the transverse direction [37]. This gene was found to be responsible for grain appearance by altering cell divisions, thereby having significant effects on regulating grain weight, as well. 

*GW8* (*GRAIN WIDTH 8*) has been reported as a positive regulator of cell proliferation and has a positive association with seed width and seed weight [31]. It encodes SQUAMOSA promoter-binding protein-like 16 (AsSPL16), that was discovered to regulate the expression of several genes involved in G1-to-S transition, similar to the regulatory role of the *GS5* gene [31,32]. It was revealed that a higher expression of the *GW8* gene promoted cell division and grain filling, resulting in increased grain width and a higher grain yield. 

*GS7* (*GRAIN SHAPE 7*), a robust QTL known to regulate grain shape, has been reported [30] to control the grain length, roundness (thickness), and area (size) in rice. Likewise, another gene, *qPE9~1*, also known as *DEP1* (*DENSE AND ERECT PANICLE 1*), encodes a G protein γ subunit found to be involved in the regulation of erect panicles, grains per panicle, nitrogen uptake, and stress tolerance through a G protein signal pathway [17,38]. In another study, the protein was also found to regulate plant architecture, grain size, and grain yield in rice. The qPE9–1 protein contains an N-terminal G gamma-like (GGL) domain, a putative transmembrane domain, and a C-terminal cysteine-rich domain [39]. Overexpression of protein qPE9–1 has been found to be responsible for increased grain size and yield in rice.

In the past few years, PCR based InDel markers have gained popularity in diversity studies because of their reproducibility, ease of use, and co-dominant inheritance [40]. InDel markers have been extensively utilized as powerful phylogenetic markers for mapping and other genetic studies in different crops [41,42,43,44,45,46,47,48]. Here, based on deletion insertion polymorphisms (DIPs), InDel markers were deployed successfully to study marker trait association and genetic variations. InDels are becoming more famous, as their genotyping requires a low start-up cost, and because they are efficient, relatively simple, and applicable to a wide range of species for which expressed sequence tag (EST) collections are available. Therefore, the leading goals of this study were to assess the efficacy of InDels to (1) estimate the population structure, allelic frequencies, and genetic variation in diverse germplasms comprising 204 rice genotypes; (2) sort the germplasms based on the distribution of the InDel marker loci; (3) assess the allele based contribution of the target genes and their association with individual traits; (4) and engagement of InDel markers to understand the genetics of traits for efficient breeding [49,50]. 

## 2. Results 

### 2.1. Descriptive Statistics and Phenotypic Variability for Rice Grain Size and Weight

Descriptive statistics (Table 1) were determined for all four studied traits, i.e., grain length (GL), grain thickness (GT), grain width (GW), and thousand grain weight (TGW), to elaborate the phenotypic variations of the respective traits in 204 rice germplasms. Collected data of whole studied germplasm for each trait is given in Table S1 (as Supplementary Material) The average values (Mean ± Standard Error) of 204 rice genotypes for GL, GW, GT, and TGW were observed to be 8.162 ± 0.065 mm, 2.932 ± 0.019 mm, 2.156 ± 0.012 mm, and 25.858 ± 0.199 g, respectively. The germplasm consisting of 204 rice genotypes showed an appreciable range for the estimated GL: 4.640 (ranging from 6.01 to 10.65 mm). Conversely, GW and GT showed lower range values (i.e., 1.800 and 1.000, respectively), ranging from 2.05 to 3.85 mm and 1.86 to 2.86 mm, respectively. Likewise, TGW was found to have an substantial range of 20 (ranging from 17 g to 37 g), depicting a wide range of variation also suggested by the higher value of variance (i.e., 8.070 in the studied germplasm). On other hand, the variance for GL (0.864) was recorded to be higher than the variance of GW and GT (having a variance of 0.073 and 0.027, respectively) (Table 1). The coefficient of variation (CV%) for all the studied traits (i.e., GL, GW, GT, and TGW) was 11.4%, 9.2%, 7.7%, and 11%, respectively. Kurtosis and skewness both symbolize the modes of gene action [51], and estimate the gene numbers controlling the trait [52], respectively. Estimated values of the skewness and kurtosis for all the studied traits are given in Table 1. Skewness was observed for GL, GT, and TGW, with values of 0.514, −0.148, 1.075, and 1.501, while the estimated kurtosis values were −0.511, 1.092, 1.965, and 1.590, respectively (Table 1). 

**Figure 2:**
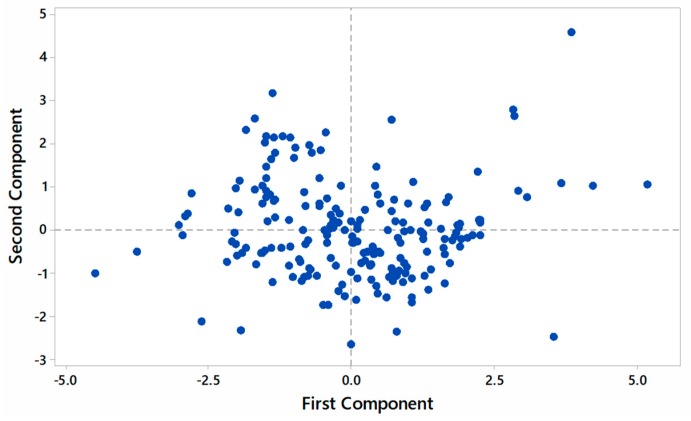
Score plot showing variability within the germplasms of 204 rice germplasms on a biplot using principal component analysis, with the first two components representing the maximum proportion (87.9%) of the total phenotypic variation for the studied traits.

Figure 2 shows the score plot showing phenotypic variability within the germplasm on a biplot using principal component analysis (PCA), with the first two components representing the maximum proportion (PC1 = 56.8%, PC2 = 31.1%) of the total variation. This shows that sufficient phenotypic variation is present in the germplasm to study genetic variation in the germplasm [53]. 

**Figure 3:**
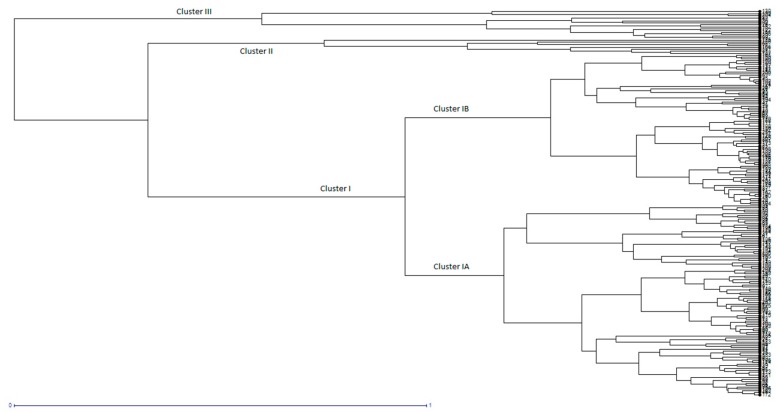
UPGMA Dendrogram showing variability in 204 rice germplasms in three distinct clusters for the studied traits estimated on the similarity index using the Euclidean distances between the groups.

Based on clustering, 204 germplasms were classified into three distinct clusters (I, II, III), as depicted in Figure 3. The major cluster, i.e., Cluster I, consisted of 182 genotypes. Cluster II consisted of 8 genotypes, whereas Cluster III had only 14 genotypes. Cluster I was further subdivided into Cluster IA and Cluster IB for simplification. Cluster IA consisted of 78 entries, and Cluster IB contained 104 entries of germplasm. The average grain length of Cluster I was calculated to be 8.01 mm, whereas the average GW and GT were 2.96 mm and 2.16 mm, respectively. The thousand grain weight (TGW) of this group was 25.63 g. Cluster II showed the highest values for the average GL (8.83 mm), indicating that the entries of this group had the maximum grain length. This group also had the maximum GT (2.27 mm), GW (3.02 mm), and the heaviest grain, as indicated by its TGW value (i.e., 30.43 g). Cluster III contained genotypes with a medium grain length (8.25 mm), but their GW (2.71 mm), GT (2.03 mm), and TGW (21.70 g) were the lowest among the groups [54].

### 2.2. Factor Analysis and Phenotypic Correlation among the Four Traits for Rice Grain Size and Weight

**Figure 4:**
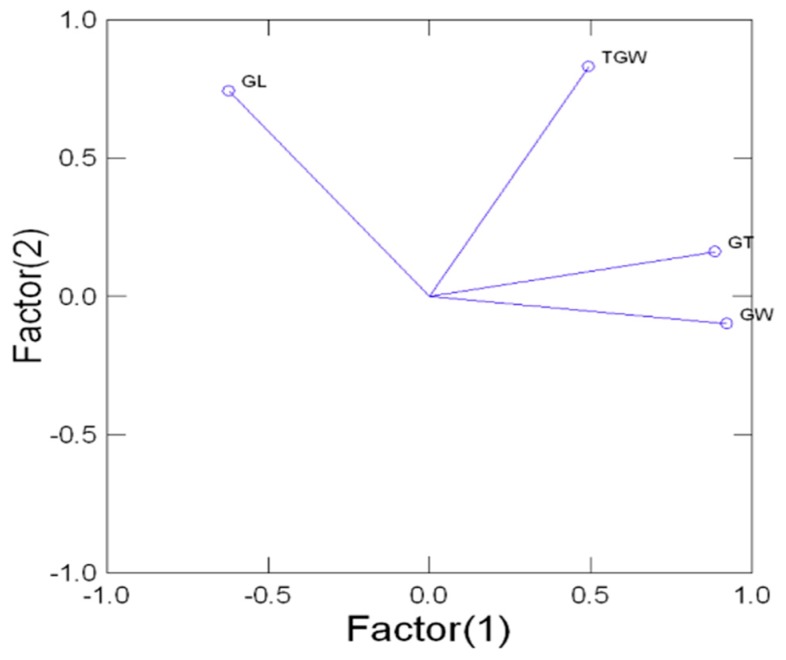
Factor analysis of the four grain size and weight related traits in rice germplasm comprising 204 genotypes; the first two factors represent more than 85% of the total variation. Traits with a positive association have vectors with an acute angle (<90) between their vectors and are closer to one another, while traits with negative associations have an obtuse (>90) angle between their vectors and are found far from one another in the biplot.

The Pearson correlation coefficients (r) were estimated for pair-wise analysis among the studied traits (i.e., GL, GW, GT, and TGW) using diverse germplasms comprising 204 rice germplasms (Table 2). A correlation analysis between GL and GW showed a highly significant (*p* ≤ 0.01) but negative association (r = −0.604 **) between these two traits. Such a negative linear relationship has also been reported by other researchers [20,55]. GL and GT were also negatively correlated (r = −0.398 *), which was also significant (*p* ≤ 0.05) and strong. In the case of TGW, GL showed a significant (*p* ≤ 0.05) and positive association (r = 0.249 *), depicting the linear relationship between these two traits. A highly significant (*p* ≤ 0.01) and positive relationship (r = 0.686 **) was also observed between GW and GT, indicating that GW was 68.6% positively influenced by GT in the studied rice germplasm. Likewise, GW showed a significant (*p* ≤ 0.05) and positive association (r = 0.374 *) with TGW. GT was also found to have a highly significant (*p* ≤ 0.01) and positive association (r = 0.491 **) with TGW. These results further revealed that TGW was 24.9% influenced by GL, 37.4% influenced by GW, and 49.1% influenced by GT. Hence, we assume the trend of trait selection (i.e., GT > GW> GL) for improving TGW in yield enhancement programs. The same results were also confirmed using Factor analysis in the Minitab software by producing a biplot for all the traits to further study the pattern of association among the traits. The results confirmed the importance of GT followed by GW and GL in improving TGW and paddy yield. Figure 4 shows a two-dimensional (2D) representation of the associations among the traits [56,57,58]. 

### 2.3. InDel Based Estimation of the Allelic Distribution of Fifteen Genes in the Determination of Grain Size and Weight 

**Figure 5:**
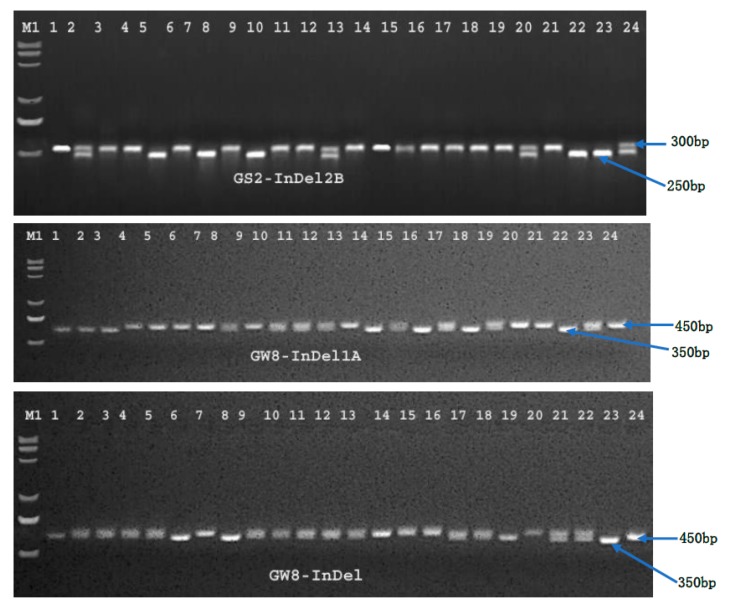

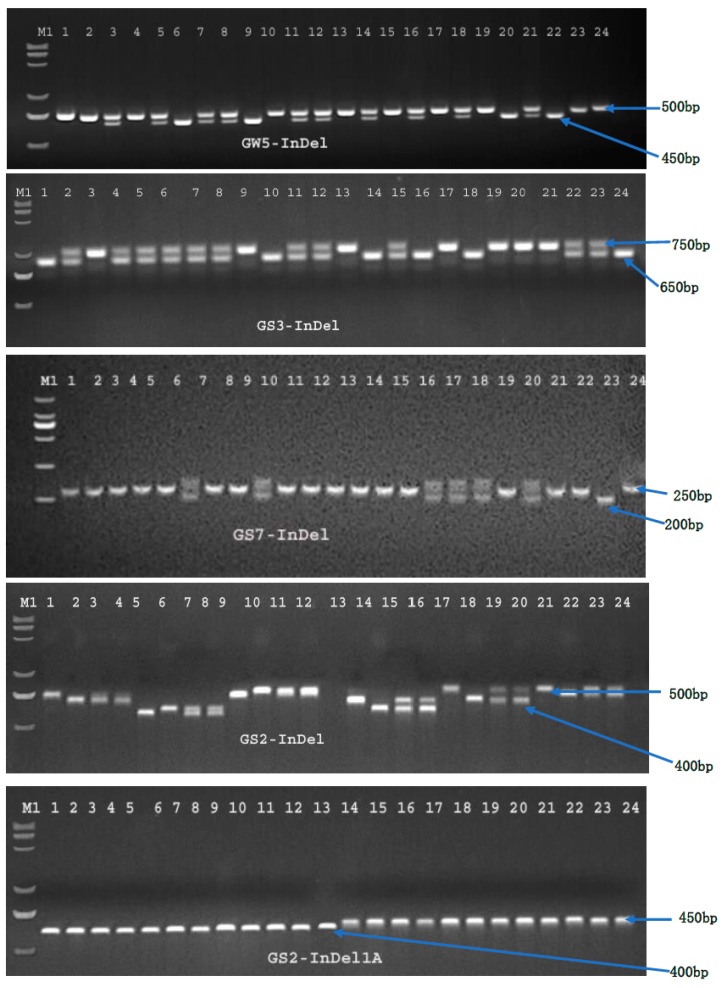

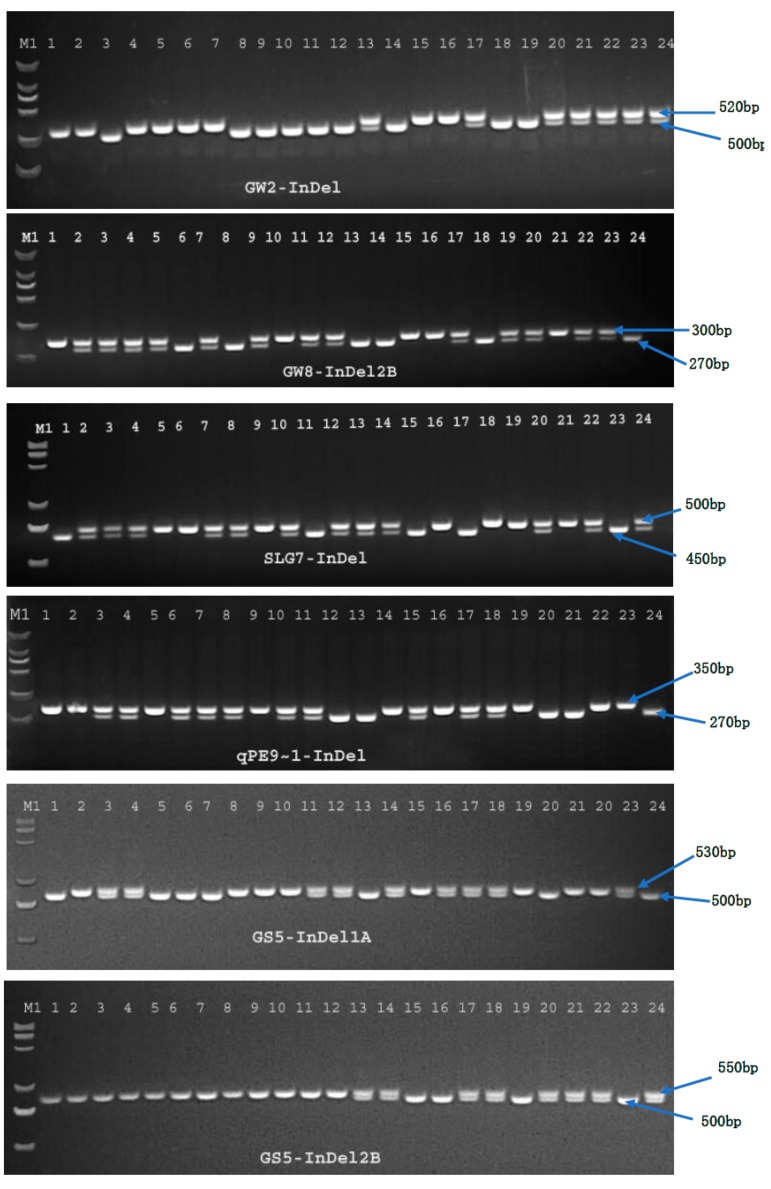
Amplification profile of the 14 Insertion/Deletion (InDel) markers of nine genes related to grain size and weight in 204 rice germplasms. M1 represents a 2000 base pair (bp) DNA ladder, 1–24 represent the rice germplasm. The size of the DNA fragment is depicted on the right side of the gel picture.

A number of genes and their alleles in different combinations have been found to be involved in determining the ultimate size, shape, and weight of rice grains (also detected by previous researchers) [7,8,9,10,11,12,13,14,15,16,17,18,19,20]. An amplification profile of 14 Insertion/Deletion (InDel) markers of nine studied genes related to grain size and weight in 204 rice germplasms is given in Figure 5. A functional insertion–deletion (InDel) marker (sequences of reverse and forward primers are given in Table 8) was used to determine the allelic frequency of the *qPE9~1* gene in the germplasm. The InDel marker produced 270 bp and 350 bp fragments, which corresponded to the A- and B-allele, respectively (Figure 5). The results (Table 3) showed that A- and B-alleles were distributed in the germplasm with frequencies of 0.8922 and 0.1078 (i.e., ~89% and ~11% of the whole germplasm) in 364 and 44 genotypes, respectively. The frequencies of alleles in the genotypes are given in Table 3. The germplasm with an A-allele of this gene was observed to have a lower grain length (7.94 ± 0.920 mm), whereas the B-allele was found to control grain length (Table 4) in rice grains, as suggested by the longer grain length (8.24 ± 0.961 mm) in the germplasm possessing the allele. Entries with lower grain widths (2.95 ± 0.275 mm) retained the A-allele, whereas the B-allele was retained by germplasm with higher values of gain width (3.05 ± 0.209 mm). For the *GW2* gene, another InDel marker (Table 8) was used to determine its allelic distribution in the germplasm. The A-allele (labelled for the fragment at 500 bp) was present in 290 rice genotypes (with 0.7108 allelic frequency), whereas the B-allele (labelled for the fragment at 520 bp) was present in 116 entries, with an allelic frequency of 0.2843 (Table 3). The A-allele was observed in the germplasm having grains with a lower grain width (2.95 ± 0.273 mm), whereas the B-allele was found in the germplasm with more grain width (3.02 ± 0.253 mm). However, the results for the gene associations with any traits were non-significant. 

The InDel marker *SLG7*-InDel (Table 8) was used to determine the allelic distribution of *SLG7* in the 204 rice germplasms. Two alleles (A and B, designated for bands at 450 bp and 500 bp, respectively) successfully divided all the germplasms into two groups: the A-allele group (308 entries) and the B-allele group (100 entries), with allelic frequencies estimates of 0.7549 (~75% of the total population) and 0.2451 (~24% of the total population), respectively (Table 3). It was further shown that both the alleles have no effect in regulating grain shape (i.e., GL, GT, and GW) (Table 4). The results depicted in Table 4 show that the gene is significantly (*p* ≤ 0.05) associated with TGW. Two alleles of the *GW5* gene, the A- and B-alleles (DNA fragments at 450 bp and 500 bp, respectively), were distinguished by another insertion–deletion (InDel) marker, *GW5*-InDel (Table 8), used to investigate the allelic frequencies of both alleles in the target germplasms of 204 rice lines. Both alleles were found in germplasm with different frequencies (Table 3). The A-allele was present in 294 genotypes, and the B-allele was found in 114 genotypes, with estimated frequencies of 0.7206 and 0.2794, respectively. Notably, the gene was found to have a significant (*p* ≤ 0.05) contribution in controlling GW and TGW. Two alleles (the A-allele labelled for the DNA fragment at 650 bp and the B-allele at 750 bp) were observed for the *GS3* gene by using an InDel marker, *GS3*-InDel (Table 8), which distinguished all the germplasms into groups, with A- and B-alleles in 204 germplasms with the frequencies of 69.6% and 30.4%, respectively, in 284 and 124 rice genotypes (Table 3). The gene was also found to have a significant (*p* ≤ 0.05) association (Table 4) with GL and GT, as indicated in Table 4, which shows that the A-allele of this gene was found in the germplasm with a less average GL (7.90 ± 0.926 mm) and thicker (2.14 ± 0.166 mm) grains, while the B-allele was present in genotypes with longer grains (8.16 ± 0.917 mm) and a low average GT (2.11 ± 0.159). The *GS7*-InDel marker (Table 8) was used to determine allelic frequencies in the subject population for the *GS7* gene. This InDel marker divided all germplasms into three groups: a group containing A-alleles and B-alleles only and a third one possessing both A- and B-alleles, which were distributed among the germplasms with different frequencies (0.4412, 0.3088, and 0.2451 respectively), present in 90, 63, and 50 entries, respectively (Table 3). It was further observed that the A-allele (DNA band labelled at 200 bp) was present in 56.4% of the total germplasm, whereas the B-allele (at 250 bp) was present in 43% of the total germplasm under study (Figure 5). Interestingly, both the A- and B-alleles showed a significantly higher mean grain length (8.19 ± 0921 and 8.08 ± 0.828 respectively) separately. However, in combination (AB), both alleles showed a lower mean grain length (7.57 ± 0.974). These studies, based on 204 germplasms, also showed that the gene has significant (*p* ≤ 0.05) associations with two grain size traits (i.e., GL and GT), indicating its contribution in regulating grain size in rice. 

An InDel marker, *GW8*-InDel (Table 8), was applied to distinguish all germplasms into two groups carrying A- and B-alleles, as designated by DNA fragments/bands at 350 bp and 450 bp, respectively (Figure 5). The A-allele was found to be present in 29.9% (122 genotypes) and the B-allele was present in the remaining 62.8% (256 genotypes) of the total germplasms (Table 3). Remarkably, the B-allele had a significantly higher mean grain length (8.41 ± 0.890), a significantly lower grain width (2.92 ± 0.259), and a lower grain thickness (2.09 ± 0.118) compared to the A-allele, which showed a lower mean grain length (7.34 ± 0.578), a significantly higher grain width (3.05 ± 0.203), and a higher thickness (2.27 ± 0.133) in the studied 204 germplasms. Based on the *GW8*-InDel marker loci in the *GW8* gene, the gene was found to be strongly associated (*p* ≤ 0.01) with three grain size traits (i.e., GL, GW, and GT) (Table 4). However, no allelic associations were observed in the case of TGW in this study. Likewise, another InDel marker, *GW8*-InDel1A was used for the same germplasm. It also separated the whole germplasm into two groups carrying A- (350 bp) and B-alleles (450 bp), with a frequency of 31.1% (127 genotypes) and 66.4% (271 genotypes), respectively. The results show that the B-allele has a significantly higher average gain length (8.40 ± 0.901) and a lower grain width (2.91 ± 0.260) and grain thickness (2.09 ±0.118) compared to the A-allele, which showed a lower average grain length (7.42 ± 0.667), a significantly higher grain width (3.06 ± 0.231), and an average grain thickness (2.27 ± 0.169) in the studied genotypes. TGW showed no changes due to these alleles. A third InDel marker, *GW8*-InDel2B, was also used for further studies, as it separated the whole germplasm into A-allele (at 270 bp) with a lower frequency (46%) and the B-allele (at 300 bp) with frequency (51.9%) that was found in 94 and 106 genotypes out of the 204 genotypes (Figure 5). These results show that B-allele has a significantly higher grain length (8.35 ± 0.852), a lower grain width (2.92 ± 0.274), and a lower grain thickness (2.10 ± 0.129) compared to the A-allele, which showed a lower average grain length (7.64 ± 0.965) and a higher average grain width (3.00 ± 0.238) in the studied genotypes. Unlike other InDel markers, *GW8*-InDel2B also distinguished the germplasm into two groups for the thousand grain weight, with the A-allele having a significantly higher TGW (26.0 ± 2.824) and the B-allele with a lower average TGW (25.0 ± 2.611). For all three InDel markers, the same results were observed for GL, GT, and GW, showing a very strong association (*p* ≤ 0.01), whereas, in the case of TGW, only one marker (*GW8*-InDel2B) showed a strong and highly significant (*p* ≤ 0.01) association, as depicted in Table 4. A-alleles of this gene should be considered while improving the GW, GT, and TGW in rice, whereas the B-alleles should be considered when breeding for longer grains. 

Another QTL *GS5* has already been reported to regulate grain size in rice via grain filling and weight [32]. In this study, two InDel markers, *GS5*-InDel1A and *GS5*-InDel1B (Table 8), were applied to determine the contribution of the affective alleles for the *GS5* gene in the germplasm. The InDel1A marker yielded two alleles, A (500 bp) and B (550 bp), with a frequency of 181 (44.3%) and 215 (52.7%), respectively (Table 3). The other InDel marker, *GS5*-InDel2B, also yielded two alleles, A (500 bp) and B (550 bp), with a frequency of 101 (24.5%) and 295 (72.3%), respectively. Both these markers had no observable association with the grain size traits in this study, except the thousand grain weight (TGW). The B-alleles of both InDel markers (i.e., *GS5*-InDel1A and *GS5*-InDel1B) were found to control the heavier grains (Table 4), as indicated by the significantly higher average TGW values (27.0 ± 2.535 and 26.2 ± 2.899, respectively) of the genotypes possessing B-alleles, whereas the germplasm with the other allele (A-allele) showed a lower average TGW for both markers (24.0 ± 3.180 and 23.0 ± 2.729, respectively). Likewise, in the case of the *GS5*-InDel1A makers, the A-allele was found to be present in 90 germplasms with a lower average grain length (7.79 ± 0.921 mm), and B-allele was found in 107 genotypes with a longer grain length (8.16 ± 0.925 mm). For the marker *GS5*-InDel2B, the A-allele was present in only seven genotypes with shorter grains (7.40 ± 0.900 mm) and in the B-allele in the larger portion of germplasm (185 genotypes) with a longer grain length (8.24 ± 0.934 mm), as represented in Table 4. The study further revealed that there is a strong (*p* ≤ 0.05) association of the *GS5* gene for both the InDel markers with TGW.

For the *GS2* gene, three InDel markers (i.e., *GS2*-InDel, *GS2*-InDel1A, and *GS2*-InDel2B (Table 8)) were used to determine the different alleles for the *GS2* gene in the germplasm of 204 entries. Two alleles, A and B, were determined for the DNA fragments at 400 bp and 500 bp, respectively, which distinguished the whole germplasm into two groups: one having 16% (65 entries) germplasm of the total (with A-alleles) and other having 83% of the total (339 entries) (with B-alleles). However, no significant associations were detected for these alleles with regards to grain size and weight related traits, suggesting the inefficiency of this marker in distinguishing this gene in the rice germplasm under study. The other InDel marker, *GS2*-InDel1A, distinguished germplasm into two groups: one with A-alleles (designated for the band at 400 bp), comprising 52.7%, and one with B-alleles (at 500 bp), comprising 45.3% of the total germplasm. Genotypes possessing B-alleles showed a significantly higher average grain length (8.44 ± 0.908), a significantly lower grain width (2.84 ± 0.275), and a lower grain thickness (2.09 ± 0.126); whereas genotypes with A-alleles showed a significantly lower average grain length (7.66 ± 0.820), as well as wider and thicker grains compared to the rest of the germplasm. No associations were observed for the TGW marker in the present study. The third marker, *GS2*-InDel2B, also distinguished A- and B-alleles, labelled for the bands at 250 bp and 300 bp, respectively, and present in 24.7% and 72.3% of the total germplasm (Figure 5). This marker separated the genotypes into two groups: one with genotypes possessing A-alleles with a significantly higher average grain length (9.32 ± 0.951), a significantly lower grain width (2.65 ± 0.303), and a lower grain thickness (2.08 ± 0.153), and the other group consisting of genotypes with B-alleles with a significantly lower average grain length (7.81 ± 0.742) and wider and thicker grains with a higher average grain width (3.01 ± 0.201) compared to the rest of the germplasm (Table 3). This gene was found to have a strong and highly significant (*p* ≤ 0.01) association with GL, GT, and GW, thereby revealing its importance in regulating all these traits in rice grains (Table 4). *GS2* showed no association with TGW, indicating that this gene is responsible only for regulating the grain size in the studied germplasm. These results showed that, for longer grains, the B-allele should be taken into consideration for the InDel1A marker, and the A-allele should be considered for the InDel2B marker.

### 2.4. Favorable Alleles of Studied Genes for Gene Pyramiding 

In the present study, a total of 38 alleles were identified. Out of these, only 27 alleles were major alleles (genotype frequency ≥0.2). Alleles present in less than 20 genotypes were considered minor alleles. Major alleles were further classified into favorable and non-favorable categories. A total of seven alleles were found to be favorable/beneficial for improving the grain length (>8 mm), having a better TGW, followed by GT and GW, respectively (Table 5). The individual role of the nine studied genes for improving grain size (GL, GW, and GT) and weight (TGW) were estimated, and the combined impact of these favorable alleles was comprehensively analyzed. ANOVA was used to test the difference between the germplasms possessing favorable alleles and non-favorable alleles in the studied germplasms (Table 5). The frequency of the favorable alleles was recorded to be in the range of 24.7% to 66.4%, with the highest for the gene *GW8* (with marker InDel1A), followed by *GW8* (InDel), *GS7* (InDel), *GW8* (InDel2B), *GS2* (InDel1A), *GS3* (InDel), and *GS2* (InDel2B), as depicted in Figure S1. All the favorable alleles, except *GS3* (InDel) and *GW8* (InDel2B), were observed to contribute to the grain length (GL > 8 mm) and thousand grain weight (Table 5). As GL and TGW are positively correlated, these genotypes may be utilized in pyramiding the target genes for longer and heavier grains to simultaneously improve the yield and quality. Average GL of the cumulated FAs were found to be higher compared with the average GL of the cumulated N-FAs depicted in Table 5. 

### 2.5. InDel Polymorphism and Assessment of the Genetic Relationship among the 204 Rice Germplasms

To study the genetic relatedness among the studied germplasm consisting of 204 rice genotypes, fourteen (14) developed Insertion/Deletion (InDel) markers from the nine grain shape, size, and weight related genes were used. Most of the InDel markers amplified 2 bands / alleles, as depicted in Figure 5. Four markers (i.e., *qPE9~1*-InDel, *SLG7*-InDel, *GW5*-InDel, and *GS3*-InDel) showed two alleles per locus, whereas the rest of the markers showed three allele types for their respective genes (Table 5). The major allele frequency ranged from 0.5196 to 0.9069, with an average value of 0.6903. The studied markers also revealed higher gene diversity (D) ranging from 0.1730–0.5246, with an average of 0.4073, indicating that the genotypes in the studied germplasm possess a considerable range of gene variations that can be further exploited. This statement is further strengthened by the higher values of the polymorphism information content (PIC) values, which have an average value of 0.3310 (0.1653–0.4359). The maximum PIC value was observed for the marker *GW8*-InDel (0.4359), followed by *GS5*-InDel1A (0.4145), *GW8*-InDel2B (0.4023), and *GS2*-InDel1A (0.4018), as mentioned in Table 6. The Average value of the observed heterozygosity was very low (0.0193), indicating that the rice germplasm under study was mostly homozygous and had uniform lines. Among all the studied markers, *GS7*-InDel (for *GS7* gene) showed considerable heterozygosity in its results compared to the other markers. The study showed that InDel markers (i.e., *GW8*-InDel, *GW8*-InDel2B, *GS5*-InDel1A, and *GS2*-InDel1A) of the *GW8*, *GS5*, and *GS2* genes are highly informative regarding these traits and can be used to understand the genetic variations in germplasm, whereas the rest of the markers are moderate to slightly informative for these traits in this study (Table 6).

In order to understand genetic variations and distinctiveness in the selected germplasm, an un-weighted neighbor joining tree was constructed (bootstraps value of 10,000) based on a dissimilarity index calculated from allelic data in the bit data format (0 and 1 indicating the absence and presence of alleles, respectively) using the Otsuka–Ochiai coefficient [47,48]. The Formula for calculating the coefficients was

$$dij=1-\;\frac{a}{\sqrt{\left(a+b\right)\left(a+c\right)}\;}.$$

**Figure 6:**
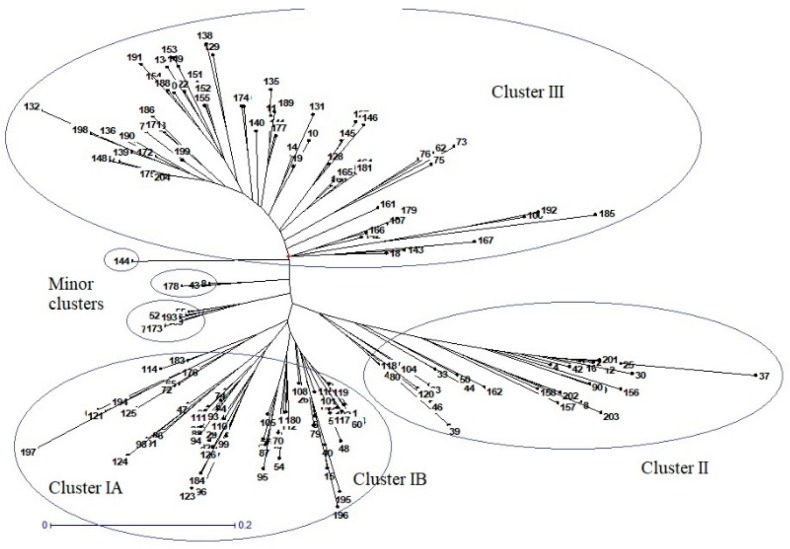
InDel marker based genetic relationship among the 204 rice germplasm entries, estimated using an un-weighted neighbor joining tree (bootstraps value of 10,000).

Based on cluster analysis, 204 germplasms were classified into six distinct clusters, encircled separately, as illustrated by the UPGMA tree in Figure 6. The three major and three minor clusters showed distinct grain characteristics. Major clusters were designated as Cluster I, Cluster II, and Cluster III. Cluster I consisted of 80 genotypes that can be further divided into two sub-clusters, IA and IB. Sub-cluster IA consists of 35 genotypes, having the highest average grain length (GL) of 9.58 mm. The average grain width (GW) and average grain thickness (GT) were 2.70 mm and 2.09 mm, respectively, whereas the thousand grain weight (TGW) of this sub-cluster was 27.07 g. Likewise, sub-cluster IB consists of 43 genotypes, with an average GL of 8.78 mm, an average GW of 2.76, an average GT of 2.07, and an average TGW of 25.52 g. The germplasm in cluster I contains extra-long grain rice genotypes that can be utilized for breeding long grain rice varieties. The major Cluster II, consisting of 35 genotypes, comprises the germplasms with an average GL, GW, GT, and TGW of 8.05 mm, 2.95 mm, 2.14 mm, and 25.36 g, respectively. This cluster contains germplasms with long and bolder grains that can be utilized in breeding programs, where grain length along with grain width and thickness are under consideration. Major Cluster III included 74 germplasms, with a mean GL of 7.38, a mean GW of 3.09 mm, a mean GT of 2.29 mm, and a mean TGW of 25.81 g, illustrating that this group consisted of rice germplasms with shorter and bolder grains. This germplasm can be utilized for short and thicker grains with a higher grain weight. The other three minor clusters contained 15 germplasms, as depicted in Figure 6.

### 2.6. Estimation of Population Genetics for Grain Size and Weight Based on Fourteen InDel Markers

**Figure 7:**
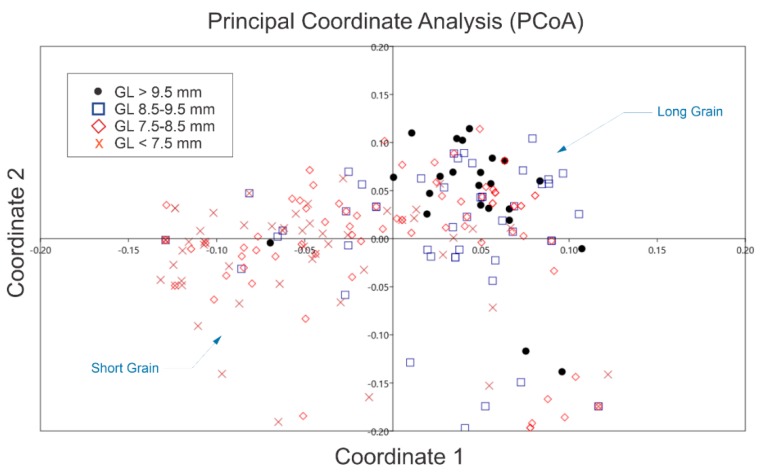
Principal Coordinate Analysis (PCoA) of 204 rice germplasms based on 14 InDel markers. A 2D scatter plot distributed the 204 germplasms based on InDel markers into four distinct groups according to the average grain length. Extra-long grains (with AGL > 9.5 mm) are shifted towards the right side, whereas short grain (AGL < 7.5 mm) germplasms shifted towards the left side. Medium grain germplasm is dispersed over the central region of the biplot.

To estimate the genetic relationship among the populations of the 204 rice genotypes in the germplasm, the total rice germplasm was divided into four sub-populations according to grain type. Extra-long grains with an average grain length (GL) of more than 9.5 mm comprising 23 rice germplasms, long grains with average AGL in the range of 8.5–9.5 mm comprising 48 rice germplasms, medium grains comprising 78 rice germplasms with an average GL between 7.5–8.5 mm, and a short grain type containing 55 germplasms with an average GL below 7.5 mm. Principal Coordinate Analysis (PCoA) was used to establish the genetic relationship of all the germplasms based on fourteen InDel markers, as depicted in Figure 7. A scatter plot was constructed with two first coordinates that collectively explain 57.3% of the total genetic variation and separate the germplasm into four types of distinct clusters according to their grain types. These results are also in agreement with the results depicted in UPGMA and the dendrogram. 

**Figure 8:**
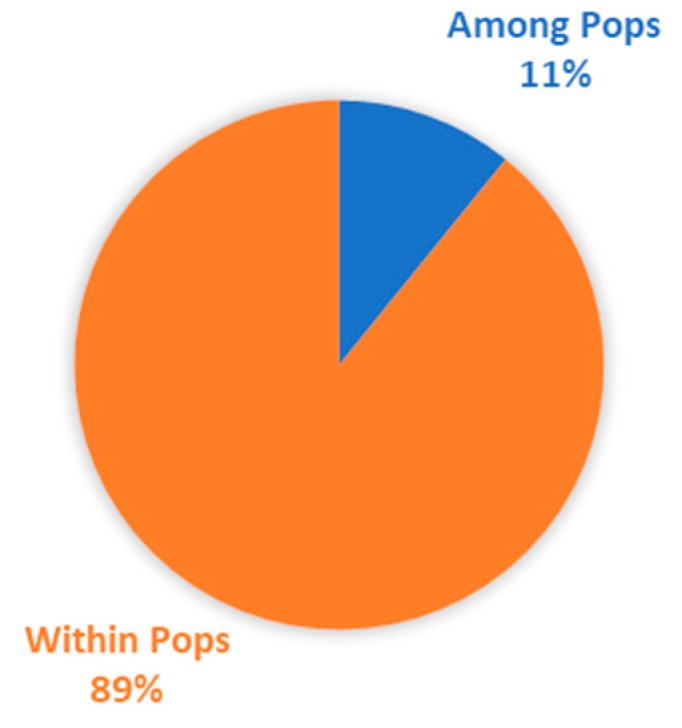
Percentages of molecular variation within and among populations.

These four sub-populations were further investigated based on the InDel marker binary data in order to separate the total molecular variance into variance within and between sub-populations. The study demonstrated that the total molecular variance was partitioned into two, of which the maximum variance (89%) was observed within the population, and the minimum was found between the populations (11%), as demonstrated in Figure 8. The results extracted from AMOVA analysis (Table 7) clearly showed that there was a maximum (Φ_PT_ = 0.449) and significant (*p* ≤ 0.001) genetic between the short grain and extra-long grain germplasms / sub-populations, while the lowest (Φ_PT_ = 0.035) genetic differentiation was observed between the long grain and medium grain sub-populations. 

## 3. Discussion

Before this study, the size and weight related traits of rice grains, including grain length, grain width, grain thickness, and thousand grain weight, had not been explored at the same time using a large and diverse germplasm group with the help of InDel markers. New Generation Sequencing (NGS) tools have yielded advanced, cheaper, and more efficient methods for developing such markers. This investigatory research efficaciously shows the capable utilization of deletion / insertion variations (DIV) that naturally accrue in the rice genome. InDel polymorphisms are the second most abundant (after SNPs) forms of genetic variations in animals and plants, with great diversity. Moreover, previous studies only showed the contributions of the reported genes in regulating these grain traits separately [11,15,16,19,20,59,60]. However, recent studies have shown the modes of InDel based allelic contributions in the expression of grain size, as well as weight related traits, the allelic combination of all the InDel marker loci involved in the final texture of the rice grains, and the potential of each loci in regulating these traits in rice. This research can assist in selecting or deselecting genes for rapid breeding strategies. The correlation coefficient values suggest that the thousand grain weight (TGW) was positively influenced by the other studied grain size traits but with a different aptitude. The TGW can be improved by using all three studied grain traits—most importantly GT, which contributes 49%, followed by GW and GL, which contribute 37.4% and 24.9% of the final grain weight, respectively. These findings are in agreement with those of previous studies [19,54,61,62], which showed that grain weight is significantly correlated with grain size. The present study further showed that the thickness of rice grains was most strongly correlated with grain weight, followed by width, whereas the length of the grains was the least associated, suggesting that selecting for grain thickness is more fruitful for heavier grains (Table 2, Figure 4). 

In the present study, two hundred and four rice genotypes were used to investigate alleles in nine different genes that regulate size and weight in rice grains, using InDel markers. In the past few years, PCR based InDel markers have gained popularity in variation studies because of their reproducibility, easy to use nature, and co-dominant inheritance [40]. Dendrograms were used to separate the germplasms according to their grain size and weight and divide the InDel marker data into distinguishable clusters that could be used for breeding preferential grain appearances and weights. Results of the genetic diversity (D) analysis and InDel based polymorphism information content (PIC) values (Table 6) indicate that the InDel markers (*GW8*-InDel, *GS5*-InDel1A, *GW8*-InDel2B, *GS2*-InDel1A) are highly informative (D ≥ 0.5; PIC ≥ 0.4) for the studied traits, whereas the rest of the markers were found to be moderately (D 0.3–0.5; PIC 0.2–0.4) to slightly/less (D ≤ 0.3; PIC ≤ 0.2) informative. These InDel markers show potential to be efficiently used to study the genetic variations (DIVs) in rice germplasm. Only two InDel markers showed very low values (gene diversity ≤0.3; PIC ≤ 0.2) for D and PIC, indicating that the deployed markers were fairly informative [20]. Furthermore, this investigatory research also showed the capability of InDel markers to distinguish a diverse rice germplasm into distinctive groups (Figure 6) with different combinations of grain lengths, widths, thicknesses, and weights that could be used to breed desirable genotypes with better potential for higher market value rice grains and heavier grains for a better yield. 

This study successfully identified 25 InDel marker derived loci highly associated (*p* ≤ 0.05) with grain size and weight in rice (Table 4). A total of 38 alleles were identified, out of which 27 alleles were major and were found in more than 20 genotypes. In the case of GL, five markers (*GW8*-InDel, *GW8*-InDel1A, *GW8*-InDel2B, *GS2*-InDel1A, and *GS2*-InDel2B) corresponding to two genes (*GW8* and *GS2*) were found to have a highly significant association with GL at *p* ≤ 0.01. Similarly, two markers (*GS3*-InDel and *GS5*-InDel1A) corresponding to two genes (*GS3* and *GS5*) were found to have a significant association with GL at *p* ≤ 0.05. In the case of GT, five markers (*GW8*-InDel, *GW8*-InDel1A, *GW8*-InDel2B, *GS2*-InDel1A, and *GS2*-InDel2B), corresponding to two genes (*GW8* and *GS2*), were found to have a significant association with GT (at *p* ≤ 0.01). Similarly, two markers (*GS3*-InDel and *GS7*-InDel), corresponding to genes *GS3* and *GS7*, were found to have a significant association with GT (at *p* ≤ 0.05). 

For GW, five markers (*GW8*-InDel, *GW8*-InDel1A, *GW8*-InDel2B, *GS2*-InDel1A, and *GS2*-InDel2B), corresponding to genes *GW8* and *GS2*, respectively, were found to have a significant association with GW at *p* ≤ 0.01. Similarly, one marker (*GW5*-InDel), corresponding to gene *GW5*, was found to have a significant association with GW at *p* ≤ 0.1. For TGW, one marker (*GW8*-InDel2B), corresponding to gene *GW8* was found to have a significant association with GW at *p* ≤ 0.01. Similarly, three markers (*GW5*-InDel, *GS5*-InDel1A, and *GS5*-InDel2B), corresponding to two genes (*GW8* and *GS5*) were found to have a significant association with GW at *p* ≤ 0.05. 

The *SLG7* gene is known to regulate the grain size in rice via increased cell division, longitudinally resulting in longer grains [21]. In the present study, the *SLG7*-InDel marker showed a significant (*p* ≤ 0.05) association with the thousand grain weight (TGW), which is also in agreement with the results of other researchers. This gene encodes the TONNEAU1-recruiting motif protein, which was found by many researchers [37] to be responsible for grain appearance by altering cell divisions, thus having significant effects in regulating grain weight, as well. Notably, GW5 gene was found to have a significant contribution in controlling GW and TGW (*p* ≤ 0.05), also revealed by previous studies [23,24,25,26], which showed that this gene encodes a calmodulin-binding protein and *GW5* physically interacts with calmodulin AsCaM1-1, which is responsible for grain width in rice. Recent studies have also demonstrated that this gene is responsible for regulating TGW (a significant correlation with *p* ≤ 0.05), as TGW is directly and highly associated with GW (Table 4), thereby confirming its utilization in grain yield improving objectives in rice breeding programs. Previous studies also identified this gene for controlling seed width and weight in rice [23,24,63].

Previous studies showed that *GS3* was among the first reported genes to have minor effects on grain thickness and width. The domains on its encoded protein have been reported to regulate cell divisions in the upper epidermis of the glume inside the rice seed, causing minor effects on cell size [29]. In the present study, the *GS3*-InDel marker was found to be significantly associated (*p* ≤ 0.05) with grain length and grain thickness, which is consistent with previous reports [20,28,31]. Another InDel marker for the *GS7* gene (*GS7*-InDel) was also found to be significantly (*p* ≤ 0.05) related with grain length and thickness. Our studies showed that the investigated alleles for both genes *GS3* and *GS7* affectively regulated grain length and thickness in the rice (Table 3). Previous studies [20,30] also reported that the germplasm carrying different alleles of the *GS3* gene with different allele combinations of *GS7* produced different grain lengths and thicknesses. Shao et al. [30] also reported that *GS7* is a strong QTL known to regulate grain size and controls grain length, roundness (thickness), and area (size) in rice. Ngangkham et al. [20] also found this gene to be associated with GL and GT, thereby playing a significant role in regulating grain size. For *GW2* gene, the results for gene associations with any trait were non-significant, but in previous studies, the gene was found to control the grain width in rice grains [12]. Ngangkham et al. [20] also found no association of this gene with any of these traits using STS (Sequence-Tagged Sites) markers, thus emphasizing the ineffectuality of the markers used. This might be due to inter- and/or intra-allelic interactions that may be subjected to further studies. 

Among all the studied genes for grain size and weight, *GW8* was detected to represent a highly significant (*p* ≤ 0.01) association with all the grain size related traits, thereby suggesting its great importance in regulating grain size in rice. The *GW8* bearing genotypes were reported to have a higher grain length and grain length-width ratio [20]. The scanning results of the electron microscopy analysis of the lemma in *GW8*.1 carrying NILs showed that the inner epidermal cell length was higher than the lines without this gene, indicating that *GW8*.1 might be responsible for regulating cell elongation [64]. *GW8* (OsSPL16) encodes a protein that is positively associated with cell proliferation [31]. Its higher expression promoted cell division and grain filling, consequentially increasing grain width and yield in rice. Another study also suggested that *GW8* suppresses the expression of the GW7 gene and plays a significant role in controlling grain size [21]. In the present study, *GW8* was determined to regulate grain length, width, and thickness. All three InDel markers successfully distinguished the germplasm into two alleles: the A-allele, which is responsible for shorter, thicker, and wider grains, and the B-allele, which carries genotypes possessing longer but narrower grains. This is due to the fact that grain length (GL) is negatively correlated with grain width (GW) and grain thickness (GT), as depicted by correlation analysis in Table 2. Based on these results, the B-allele carrying germplasm may be selected to breed longer grains, and the A-alleles may be screened for broader, shorter, and thicker grains. However, all three traits (i.e., GL, GT, and GW) contributed to the thousand grain weight (as suggested by the positive correlation between GL, GT, and GW with thousand grain weight), assuming that both alleles contribute to an increased yield. Two markers (i.e., *GW8*-InDel and *GW8*-InDel1A) were shown to have highly significant (*p* ≤ 0.0001) associations with GL, GT, and GW, thus indicating the ample potential of InDel markers in variation studies and genome-wide association studies. The third marker (i.e., *GW8*-InDel2B) for the *GW8* gene was also identified to have a highly significant (*p* ≤ 0.01) association with GL and GW (Table 5). However, unlike the other two InDel markers for the *GW8* gene, *GW8*-InDel2B also showed a highly significant (*p* ≤ 0.0001) relationship with GT and the thousand grain weight (Table 4), indicating its potential to be used for all four studied traits to improve the grain size and grain weight in rice. 

Two InDel based markers were used for the *GS5* gene, and both of these markers showed a significant (*p* ≤ 0.05) association with only the thousand grain weight. These findings are partially inconsistent with other studies [32,65], which suggested that the *GS5* gene is associated with grain width and grain weight in rice. Previously, Lee et al. [63] attained three types of alleles by applying the markers generated from the promoter-region of the *GS5* gene, thus demonstrating the relatedness of this gene with grain weight. However, in another study, this gene was reported to participate in the regulation of grain length and grain width [63]. This might be due to higher genetic and/or allelic interactions with other genes/alleles that must be studied more comprehensively. This gene has been reported to have significant importance in regulating grain yield, as concluded by Li et al. [32], who showed that this gene encoded proteins—i.e., the putative serine carboxypeptidase executes its function as a positive regulator of a subset of the transition genes (G1-to-S) of the cell cycle, thereby causing increased cell divisions and resulting in enhanced grain filling and grain weight. 

The Present study further explored the previously reported gene *GS2* to be highly associated (*p* ≤ 0.001) with all three-grain size related traits. Out of the three markers, two markers for this gene (including *GS2*-InDel1A and *GS2*-InDel2B) showed the potential for GL, GT, and GW, whereas the marker *GS2*-InDel showed no association with any trait (Table 4). For both markers 1A and 1B for this gene, the germplasm was separated into two groups carrying A- and B-alleles with different grain size traits. In the case of InDel1A, the A-allele was associated (*p* ≤ 0.001) with a shorter grain length (7.66 ± 0.820 mm) with thicker and wider grains, whereas its B-allele had a germplasm with a longer (8.44 ± 0.908 mm) grain length and narrower and slander grains (Table 5). Conversely, for the other marker, *GS2*-InDel2B, the A-allele was detected to relate to the germplasm with the longest grain lengths (9.32 ± 0.951 mm) and narrowest grains (the GW average is 2.65 ± 0.303 mm) in the whole germplasm, whereas the genotypes carrying the B-allele possessed shorter grains (7.81 ± 0.742 mm) and wider grains (3.01 ± 201 mm), as depicted in Table 4. This finding suggests that these InDel markers can further be investigated to breed for >9 mm grain lengths. Previous studies also showed that this gene directly controls two important grain size related traits, including grain width and grain length in rice. Researchers showed that its overexpression increased cell enlargement and enhanced cell division in the grain, thus producing longer and wider rice grains [33,34,35,36].

This study further investigated the favorable alleles in the studied germplasm to improve the grain length (>8 mm) with heavier grains. Identifying the beneficial alleles of the target traits is one of the most important prerequisites to improve modern cultivars via introgressions of favorable alleles from a vast gene pool using marker assisted selection approaches. This investigatory research discovered seven favorable alleles for grain length that can be utilized to improve grain size, while keeping in mind the recent criterion for longer grains with improved grain sizes and weights. 

This research explored 7 genes and 11 InDel marker associations with grain size and weight related traits in rice. The present study further showed that InDel markers may be used efficiently in research investigations related to genetic variations, genome-wide association studies, germplasm genetic characterization, gene mapping, and other studies to further develop the ease and efficiency of breeding procedures and create more desirable varieties to cope with climate change and food security risks. 

## 4. Materials and Methods

### 4.1. Rice Material Collection and Phenotyping

The plant material of this experiment included 204 rice germplasms, which included lines with a wide range of grain size related traits. Plant material was received from the Key Laboratory of Crop Genetics and Breeding section, Yangzhou University, Jiangsu province, P.R. China, comprising rice lines with higher yields. The field experiment was carried out in the experimental fields of the research capacity in Yangzhou University (E, N) during the normal rice season (from May to November) in 2018. Sowing of the material was carried out in the nursery for the grain cell, and then the 30 day old nursery was transplanted into well prepared fields following standard agronomic practices in order to assure full expression of the traits. Plant to Plant distance was maintained at 10 cm, whereas row to row distance was maintained at 15 cm. For grain trait phenotyping, data were collected during the stage of plant maturity from the middle plants in the central row of each entry. Grain size related traits (i.e., grain length (GL), grain width (GW), and grain thickness (GT)) were measured in (millimeters) as the average from ten completely mature and filled grains using digital Vernier calipers. Likewise, data for grain weight related traits (i.e., thousand grain weight (TGW)) were measured for 250 carefully counted, filled, and fully mature rice grains using an electronic digital weighing balance, and the values were multiplied by a factor of 4 to obtain the 1000 grain weight. 

### 4.2. DNA Extraction and PCR

DNA was extracted from fresh rice leaves according to the method described in [66], with minor modifications. PCR (polymerase chain reaction) was conducted, and the products were separated by agarose gel electrophoresis [67]. A set of newly developed insertion/deletion (InDel) markers was selected as the primary markers, as found in the open rice genome sequence library (http://www.ncbi.nlm.nih.gov). Amplified DNA products were analyzed on 3% agarose gels stained with ethidium bromide and photographed with a UVP system.

### 4.3. Marker Genotyping 

The germplasm comprising 204 rice germplasms was mined for the presence of 9 grain size and weight related genes (i.e., *qPE9~1*, *GW2*, *SLG7*, *GW5*, *GS3*, *GS7*, *GW8*, *GS5*, and *GS2*) using the 14 InDel markers given in Table 8, along with detailed information of the primer pairs for each marker. All the markers were scored visually as 1 for their presence and 0 for their absence. 

### 4.4. Statistical Analyses

Descriptive statistics and factorial analyses for the trait association of GL, GT, GW, and TGW were conducted using the statistical package SYSTAT, version 13.1. Principal Component Analysis (PCA) for testimation of the phenotypic variability within the germplasm was performed using Minitab software, version 18. Correlation coefficients among the grain size and weight related traits were determined using the computer-based software, IBM-SPSS, version 25.

For allele scoring, the genetic variations, expected heterozygosity, and polymorphism information content (PIC) values of the 14 markers were calculated using binary data in the POWER MARKER software, version 3.25 [68]. The presence of alleles was scored as 1, whereas the absence of alleles was scored as 0 to generate a binary matrix for each genotype. An un-weighted neighbour joining UPGMA tree was built based on the marker data using the DARwin 6 software [69]. Another UPGMA dendrogram (to categorize different groups based on phenotypic data) was constructed using the PAST version 3.25 software. A scatter plot was constructed using the PAST version 3.25 software to disperse the germplasm further into the sub-populations based on the InDel marker data [63,70]. The Principal Coordinate Analysis (PCoA) analysis and Analysis of Molecular Variance (AMOVA) were conducted from the binary data of the InDel markers using GenAlEx version 6.502. The binary data of the genetic markers were constructed according to grain size, which was used to separate the total molecular variance between and within the groups. **Φ**_PT_ was also calculated using GenAlEx v. 6.502.

## 5. Conclusions

Collectively, it can be concluded that allelic variations of the nine genes are extensively distributed in the studied germplasm. Further, we observed that most of these alleles are significantly associated with variations in one or more of the studied traits related to grain size and grain weight in rice. The results also suggest that several genotypes have similar grain size characteristics and share particular allele and/or allele combinations of the nine key genes examined in this study. The examinations of allelic contributions from different genes in regulating grain size and weight related traits undertaken in the study will further strengthen our understanding of the complex mechanisms involved in rice grain appearance and the grain weight to be utilized in rice breeding programs. The identified genes and their linked InDel markers could be highly informative in pyramided breeding strategies and selecting parental lines for developing rice varieties according to consumer needs. 

## Figures and Tables

**Table 1:** Descriptive statistics for grain length (GL), grain width (GW), grain thickness (GT), and thousand grain weight (TGW) of germplasms containing two hundred and four (204) rice germplasms.

Parameters	GL (mm)	GW (mm)	GT (mm)	TGW (g)
Minimum	6.010	2.050	1.860	17.0
Maximum	10.650	3.850	2.860	37.0
Range	4.640	1.800	1.000	20.0
Arithmetic Mean	8.162	2.932	2.156	25.858
Standard Error of Arithmetic Mean	0.065	0.019	0.012	0.199
Standard Deviation	0.929	0.270	0.165	2.841
Variance	0.864	0.073	0.027	8.070
Coefficient of Variation (CV)	0.114	0.092	0.077	0.110
Skewness (G1)	0.514	−0.148	1.075	1.501
Kurtosis (G2)	−0.511	1.092	1.965	1.591

**Table 2:** Correlation coefficient analysis among the four studied traits for grain size and weight.

Traits	GL	GW	GT
Grain length (GL)	1.000		
Grain width (GW)	−0.604 **	1.000	
Grain thickness (GT)	−0.398 *	0.686 **	1.000
Thousand grain weight (TGW)	0.249 *	0.374 *	0.491 **

** highly significant at *p* ≤ 0.01; * significant at *p* ≤ 0.05.

**Table 3:** Fragment lengths on electrophoresis gel, expected frequency estimates, allelic variance, and standard deviation (SD) estimates of each allele of the 14 InDel loci.

Marker	Allele	Band Length (bp)	Genotype	Expected Frequency	Variance	SD
*qPE9~1*-InDel	A	270	364	0.8922	0.0005	0.0217
	B	350	44	0.1078	0.0005	0.0217
*GW2*-InDel	A	500	290	0.7108	0.0010	0.0317
	B	520	116	0.2843	0.0010	0.0316
*SLG7*-InDel	A	450	308	0.7549	0.0009	0.0301
	B	500	100	0.2451	0.0009	0.0301
*GW5*-InDel	A	450	294	0.7206	0.0010	0.0314
	B	500	114	0.2794	0.0010	0.0314
*GS3*-InDel	A	650	284	0.6961	0.0010	0.0322
	B	750	124	0.3039	0.0010	0.0322
*GS7*-InDel	A	200	230	0.5637	0.0009	0.0301
	B	250	176	0.4314	0.0009	0.0300
*GW8*-InDel	A	350	122	0.2990	0.0010	0.0321
	B	450	256	0.6275	0.0011	0.0339
*GW8*-InDel1A	A	350	127	0.3113	0.0010	0.0323
	B	450	271	0.6642	0.0011	0.0330
*GW8*-InDel2B	A	270	188	0.4608	0.0012	0.0349
	B	300	212	0.5196	0.0012	0.0350
*GS5*-InDel1A	A	500	181	0.4436	0.0012	0.0347
	B	530	215	0.5270	0.0012	0.0349
*GS5*-InDel2B	A	500	14	0.0343	0.0002	0.0127
	B	550	370	0.9069	0.0004	0.0203
*GS2*-InDel	A	400	65	0.1593	0.0007	0.0255
	B	500	339	0.8309	0.0007	0.0261
*GS2*-InDel1A	A	400	215	0.5270	0.0012	0.0349
	B	450	185	0.4534	0.0012	0.0348
*GS2*-InDel2B	A	250	101	0.2475	0.0009	0.0301
	B	300	295	0.7230	0.0010	0.0312

**Table 4:** Allelic variations of the grain size and weight regulating genes in 204 rice germplasms, based on InDel marker loci.

Markers	Alleles	Germplasm	Grain Length (mm)		Grain Width (mm)		Grain Thickness (mm)		Thousand Grain Weight (g)	
			Mean ± SD	*p* Value	Mean ± SD	*p* Value	Mean ± SD	*p* Value	Mean ± SD	*p* Value
*qPE9~1*-InDel	A	182	7.94 ± 0.920 ^b^	0.32	2.95 ± 0.275 ^b^	0.49	2.13 ± 0.167 ^a^	0.86	26.0 ± 2.837 ^a^	0.32
	B	22	8.24 ± 0.961 ^a^		3.06 ± 0.209 ^a^		2.12 ± 0.149 ^a^		26.5 ± 2.672 ^a^	
*GW2*-InDel	A	145	7.98 ± 0.934 ^a^	0.56	2.95 ± 0.273 ^b^	0.17	2.13 ± 0.167 ^a^	0.47	26.0 ± 2.984 ^a^	0.52
	B	58	8.03 ± 0.907 ^a^		3.02 ± 0.253 ^a^		2.12 ± 0.160 ^a^		26.0 ± 2.441 ^a^	
*SLG7*-InDel	A	154	7.97 ± 0.933 ^a^	0.89	2.95 ± 0.268 ^a^	0.98	2.12 ± 0.160 ^a^	0.23	26.0 ±2.857 ^a^	0.027 *
	B	50	8.03 ± 0.907 ^a^		2.99 ± 0.273 ^a^		2.16 ± 0.178 ^a^		26.0 ± 2.695 ^a^	
*GW5*-InDel	A	147	7.98 ± 0.894 ^a^	0.93	2.95 ± 0.261 ^a^	0.047 *	2.13 ± 0.163 ^a^	0.29	25.0 ± 2.847 ^b^	0.031 *
	B	57	7.93 ± 1.008 ^a^		3.00 ± 0.285 ^a^		2.13 ± 0.170 ^a^		26.0 ± 2.708 ^a^	
*GS3*-InDel	A	142	7.90 ± 0.926 ^a^	0.019 *	2.97 ± 0.279 ^a^	0.44	2.14 ± 0.166 ^a^	0.012 *	26.0 ± 2.729 ^a^	0.61
	B	62	8.16 ± 0.917 ^a^		2.95 ± 0.244 ^a^		2.11 ± 0.159 ^a^		25.5 ± 3.054 ^a^	
*GS7*-InDel	A	90	8.19 ± 0.921 ^a^	0.022 *	2.95 ± 0.299 ^a^	0.56	2.10 ± 0.162 ^b^	0.0194 *	26.0 ±2.889 ^a^	0.96
	B	63	8.08 ± 0.828 ^a^		2.97 ± 0.190 ^a^		2.12 ± 0.162 ^b^		26.0 ± 2.504 ^a^	
	AB	50	7.57 ± 0.974 ^b^		2.95 ± 0.293 ^a^		2.20 ± 0.159 ^a^		25.0 ± 3.116 ^b^	
*GW8*-InDel	A	60	7.34 ± 0.578 ^b^	≤0.0001 *	3.05 ± 0.203 ^b^	≤0.0001 *	2.27 ± 0.133 ^a^	≤0.0001 *	25.0 ± 2.694 ^b^	0.06
	B	128	8.41 ± 0.890 ^a^		2.92 ± 0.259 ^a^		2.08 ± 0.118 ^b^		26.0 ± 2.583 ^a^	
*GW8*-InDel1A	A	63	7.42 ± 0.667 ^b^	≤0.0001 *	3.06 ± 0.231 ^b^	≤0.0001 *	2.27 ± 0.169 ^a^	≤0.0001 *	26.0 ± 3.309 ^a^	0.0620
	B	135	8.40 ± 0.901 ^a^		2.91 ± 0.260 ^a^		2.09 ± 0.118 ^b^		26.0 ± 2.615 ^a^	
*GW8*-InDel2B	A	94	7.64 ±0.965 ^b^	0.0045 *	3.00 ± 0.238 ^a^	0.003 *	2.19 ± 0.158 ^a^	≤0.0001 *	26.0 ± 2.824 ^a^	≤0.0001 *
	B	106	8.35 ± 0.852 ^a^		2.92 ± 0.274 ^b^		2.10 ± 0.129 ^b^		25.0 ± 2.611 ^b^	
*GS5*-InDel1A	A	90	7.79 ± 0.921 ^b^	0.12	2.97 ± 0.259 ^a^	0.75	2.13 ± 0.165 ^a^	0.22	24.0 ± 3.180 ^b^	0.016 *
	B	107	8.16 ± 0.925 ^a^		2.94 ± 0.272 ^a^		2.12 ± 0.150 ^a^		27.0 ± 2.535 ^a^	
*GS5*-InDel2B	A	7	7.40 ± 0.900 ^b^	0.87	2.78 ± 0.261 ^b^	0.33	2.14 ± 0.161 ^a^	0.44	23.0 ± 2.729 ^b^	0.038 *
	B	185	8.24 ± 0.934 ^a^		2.99 ± 0.265 ^a^		2.21 ± 0.161 ^b^		26.2 ± 2.899 ^a^	
*GS2*-InDel	A	32	7.57 ± 1.035 ^a^	0.96	3.03 ± 0.314 ^a^	0.99	2.22 ± 0.180 ^a^	0.18	27.0 ± 3.258 ^a^	0.58
	B	169	7.98 ± 0.900 ^a^		2.94 ± 0.261 ^b^		2.12 ± 0.161 ^b^		25.0 ± 2.729 ^b^	
*GS2*-InDel1A	A	107	7.66 ± 0.820 ^b^	≤0.0001 *	3.01 ± 0.246 ^a^	0.0005 *	2.19 ± 0.180 ^a^	≤0.0001 *	26.0 ± 2.943 ^a^	0.89
	B	92	8.44 ± 0.908 ^a^		2.84 ± 0.275 ^b^		2.09 ± 0.126 ^b^		26.0 ± 2.754 ^a^	
*GS2*-InDel2B	A	50	9.32 ± 0.951 ^a^	≤0.0001 *	2.65 ± 0.303 ^b^	≤0.0001 *	2.08 ± 0.153 ^b^	0.0074 *	26.0 ± 3.136 ^a^	0.12
	B	147	7.81 ± 0.742 ^b^		3.01 ± 0.201 ^a^		2.16 ± 0.155 ^a^		25.0 ± 2.761 ^b^	

Data present the mean values ± standard deviation; ANOVA test for significant at the level of *p* level <0.05 and <0.01. * significant at *p* ≤ 0.05. ^a, b^ and ^c^ were ranked by Duncan’s test.

**Table 5:** Pyramiding of the favorable alleles of the studied genes for Grain Length (GL).

Genes	Markers	Alleles	Grain Length (mm)		Grain Width (mm)		Grain Thickness (mm)		Thousand Grain Weight (g)	
			FA (Mean ± SD)	N-FA (Mean ± SD)	FA (Mean ± SD)	N-FA (Mean ± SD)	FA (Mean ± SD)	N-FA (Mean ± SD)	FA (Mean ± SD)	N-FA (Mean ± SD)
*GS3*	*GS3*-InDel	A	-	7.90 ± 0.926 ^a^	2.97 ± 0.279 ^a^	-	-	-	-	-
		**B**	8.16 ± 0.917 ^a^	-	-	2.95 ± 0.244 ^a^	-	-	-	25.5 ± 3.054 ^a^
*GS7*	*GS7*-InDel	**A**	8.19 ± 0.921 ^a^	-	-	-	-	-	26.0 ± 2.889 ^a^	-
		**B**	8.08 ± 0.828 ^a^	-	-	2.97 ± 0.190 ^a^	-	2.12 ±0.162 ^b^	-	26.0 ± 2.504 ^a^
*GW8*	*GW8*-InDel	A	-	7.34 ± 0.578 ^b^	-	3.05 ± 0.203 ^b^	-	2.27 ± 0.133 ^a^	-	25.0 ± 2.694 ^b^
		**B**	8.41 ± 0.890 ^a^	-	-	-	-	-	26.0 ± 2.583 ^a^	-
	*GW8*-InDel1A	A	-	7.42 ± 0.667 ^b^	-	3.06 ± 0.231 ^b^	-	2.27 ± 0.169 ^a^	-	26.0 ±3.309 ^a^
		**B**	8.40 ± 0.901 ^a^	-	-	-	-	-	26.0 ± 2.615 ^a^	-
	*GW8*-InDel2B	A	-	7.64 ± 0.965 ^b^	-	3.00 ± 0.238 ^a^	-	2.19 ± 0.158 ^a^	-	26.0 ±2.824 ^a^
		**B**	8.35 ± 0.852 ^a^	-	-	-	-	-	-	-
*GS2*	*GS2*-InDel1A	A	-	7.66 ± 0.820 ^b^	-	3.01 ± 0.246 ^a^	-	2.19 ± 0.180 ^a^	-	26.0 ±2.943 ^a^
		**B**	8.44 ± 0.908 ^a^	-	-	-	-	-	26.0 ± 2.754 ^a^	-
	*GS2*-InDel2B	**A**	9.32 ± 0.951 ^a^	-	-	-	-	-	26.0 ± 3.136 ^a^	-
		B	-	7.81 ± 0.742 ^b^	-	3.01 ± 0.201 ^a^	-	2.16 ± 0.155 ^a^	-	25.0 ±2.761 ^b^
Mean ± SD			8.47 ± 0.39 ^a^	7.62 ± 0.22 ^b^	2.97 ± 0.279 ^a^	3.01 ± 0.030 ^a^	-	2.20 ± 0.060	26.0 ± 0.00 ^a^	25.6 ±0.51 ^b^

FA and N-FA symbolize favorable and non-favorable alleles, respectively; a and b represent significantly (*p* value < 0.01) different mean values. Sign (-) symbolizes no FA/N-FA allele under this category

**Table 6:** Estimation of the number of alleles per locus, the major allele frequency, gene diversity (D), expected heterozygosity, and the polymorphism information content (PIC) values in the 204 rice germplasms.

Marker	*A*	*p_ma_*	*D*	*H_Exp_*	PIC
*qPE9~1*-InDel	2	0.8922	0.1924	0.0000	0.1739
*GW2*-InDel	3	0.7108	0.4139	0.0000	0.3322
*SLG7*-InDel	2	0.7549	0.3700	0.0000	0.3016
*GW5*-InDel	2	0.7206	0.4027	0.0000	0.3216
*GS3*-InDel	2	0.6961	0.4231	0.0000	0.3336
*GS7*-InDel	3	0.5637	0.4961	0.2451	0.3778
*GW8*-InDel	3	0.6275	0.5115	0.0000	0.4359
*GW8*-InDel1A	3	0.6642	0.4613	0.0049	0.3752
*GW8*-InDel2B	3	0.5196	0.5173	0.0000	0.4023
*GS5*-InDel1A	3	0.5270	0.5246	0.0049	0.4145
*GS5*-InDel2B	3	0.9069	0.1730	0.0000	0.1653
*GS2*-InDel	3	0.8309	0.2842	0.0049	0.2490
*GS2*-InDel1A	3	0.5270	0.5163	0.0049	0.4018
*GS2*-InDel2B	3	0.7230	0.4151	0.0049	0.3500
Mean	3	0.6903	0.4073	0.0193	0.3310

*A* = alleles per locus; *p*_ma_ = major allele frequency; *D* = gene diversity; *H*_Exp_ = expected heterozygosity; PIC = polymorphism information content.

**Table 7:** Pair-wise population ɸ_PT_ value estimates among the four sub-populations of 204 rice germplasms.

	Extra-Long Grain	Long Grain	Medium Grain
Long grain	0.097		
Medium grain	0.212	0.035	
Short grain	0.449	0.264	0.129

Φ_PT_ values given below the diagonal; calculated for 10,000 permutations.

**Table 8:** Detailed information for the 14 molecular markers for the 9 grain size and weight related genes.

S.N.	Genes	Markers	Forward (5’ to 3’)	Reverse (5’ to 3’)	Tm(°C)	Types
1	*qPE9~1*	*qPE9~1*-InDel	AGTGGTGCCTATAACTCTGC	AGCAAAGAGGACGTCATACT	55	InDel
2	*GW2*	*GW2*-InDel	GCAATGCAAAAGCATATGGC	AAAGCCAAAGATGCACACAG	53	InDel
3	*SLG7*	*SLG7*-InDel	TGGTTTCGATTAGGTTCCTCT	AAAACGCCGTTTAGCTATCC	52	InDel
4	*GW5*	*GW5*-InDel	GAACTACATGTCCAACACGC	CTCCACCACCACCACCTC	58	InDel
5	*GS3*	*GS3*-InDel	ATGACCACGTCGATCATCAA	ACTCCACCTGCAGATTTCTT	53	InDel
6	*GS7*	*GS7*-InDel	TGGTCAAATCATGGGCTAAT	TATTATTGTGCCTGCGATCC	51	InDel
7	*GW8*	*GW8*-InDel	AAAAGAGACAGCCACGGAAT	TCTTGAGATCCCACTCCATG	54	InDel
8		*GW8*-InDel1A	AAAAGAGACAGCCACGGAAT	TCTTGAGATCCCACTCCATG	55	InDel
9		*GW8*-InDel2B	TTTCAGTGTCCTCCTGTCTG	ACCACTAAACCAGGTGCTAC	56	InDel
10	*GS5*	*GS5*-InDel1A	TGACGCCGTTGACTTTTTGA	GAATCCGGCGTTGATTTCGA	55	InDel
11		*GS5*-InDel2B	AGGTGTTGTTCGAAACTCACG	TGAAAATTTGGATATTCGTGGCA	54	InDel
12	*GS2*	*GS2*-InDel	CCCACCGCATGATACATCTA	ATGTGGGAATTTCTAGCCCC	55	InDel
13		*GS2*-InDel1A	GCCGCGGTCTTTAGTAATGG	CCTTCTCGTGTCGGGCTC	59	InDel
14		*GS2*-InDel2B	AAATTGCAGCCGACCGTAG	AAATTGTGACGAGTCCCAGC	55	InDel

## Supplementary Materials

Supplementary materials can be found at https://www.mdpi.com/1422-0067/20/19/4824/s1.
