# Supplementary material for: InDel Marker Based Estimation of Multi-Gene Allele Contribution and Genetic Variations for Grain Size and Weight in Rice (Oryza sativa L.)
Source: Int J Mol Sci. 2019 Sep 28;20(19):4824. doi: 10.3390/ijms20194824 (PMC6801599; doi:10.3390/ijms20194824)
Supplement: Supplementary file 1 [file ijms-20-04824-s001.zip › ijms-561276-figure s1.docx]

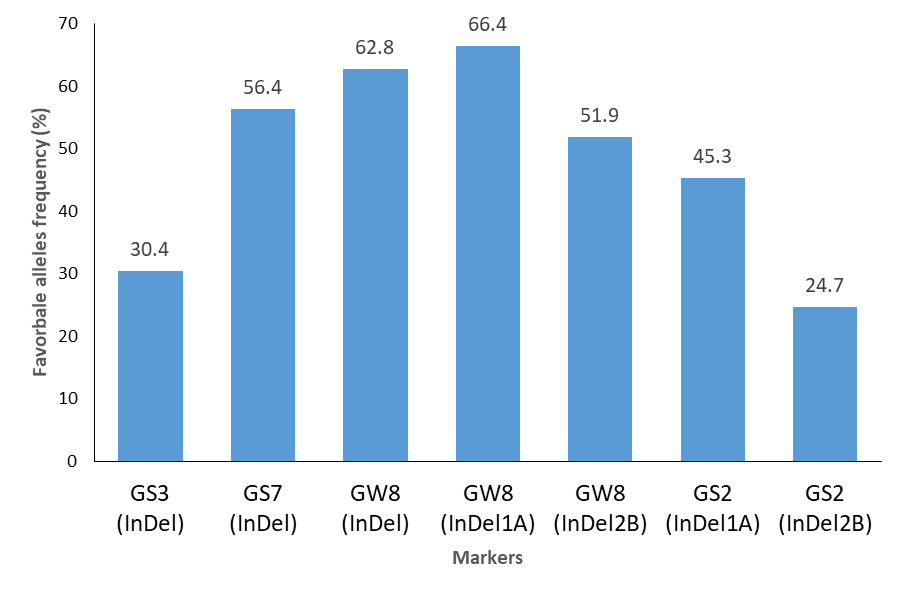


**Figure S1**. The InDel marker based distribution frequencies of the favorable alleles of seven genes related to grain size and weight in 204 rice genotypes.
